# Supplementary material for: A hybrid model based on CNN-LSTM for assessing the risk of increasing claims in insurance companies
Source: PeerJ Comput Sci. 2025 Apr 21;11:e2830. doi: 10.7717/peerj-cs.2830 (PMC12190450; doi:10.7717/peerj-cs.2830)
Supplement: Supplemental Information 1 [file peerj-cs-11-2830-s001.docx]

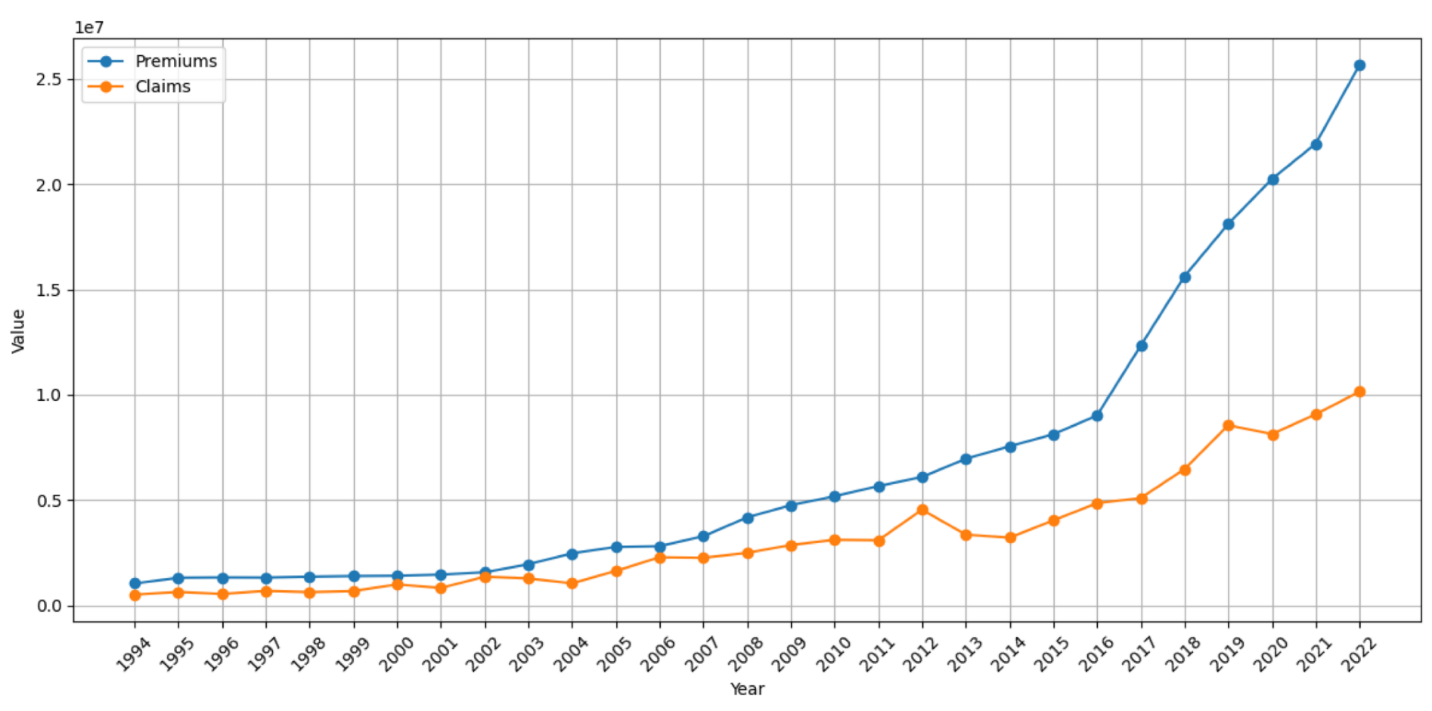


**Supplemental Figure 1:** Sample of insurance companies' evolution of premiums and claims in insurance companies

| 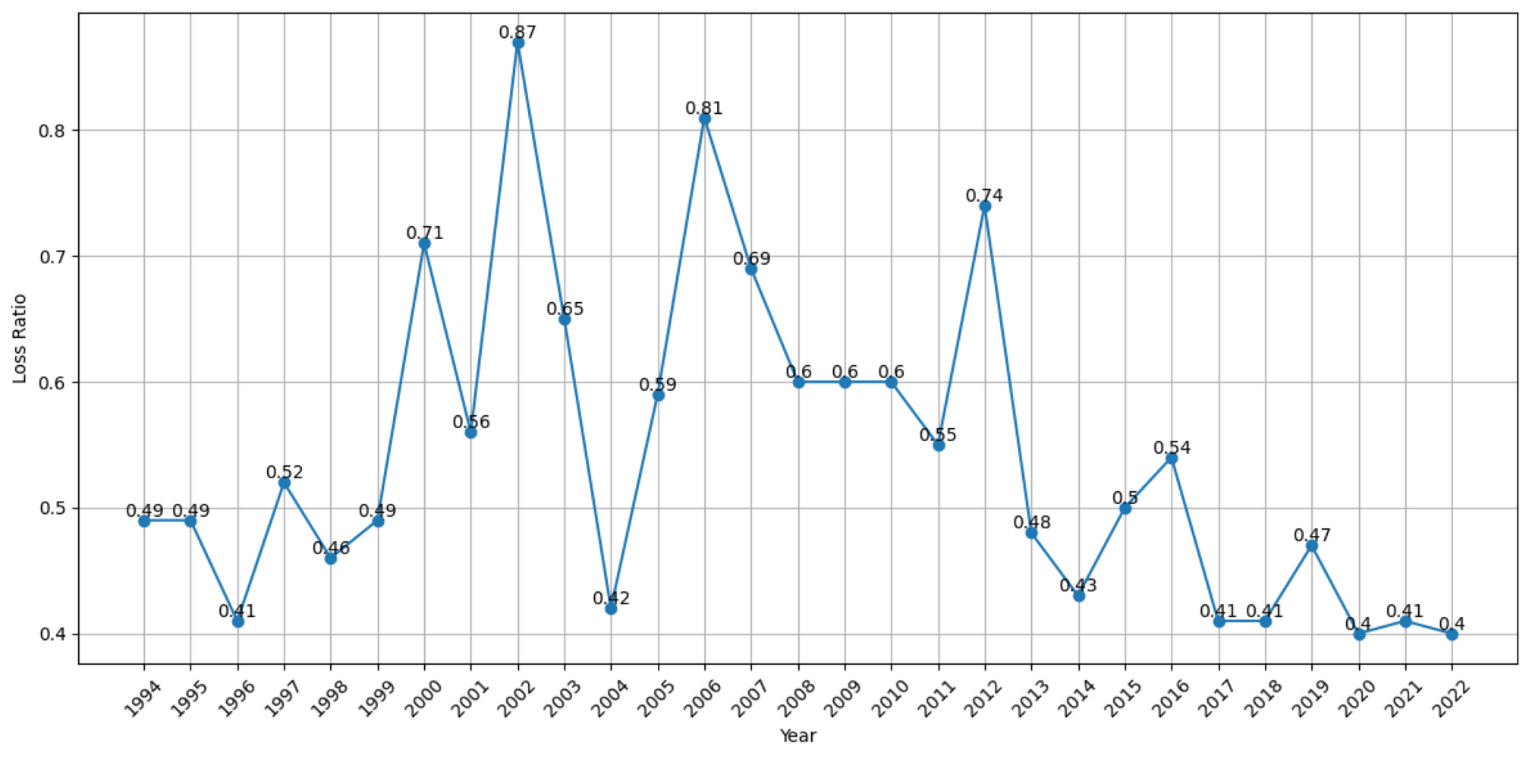 |
| --- |
| **Supplemental Figure 2:** Observed loss ratio of a risk group in insurance companies |


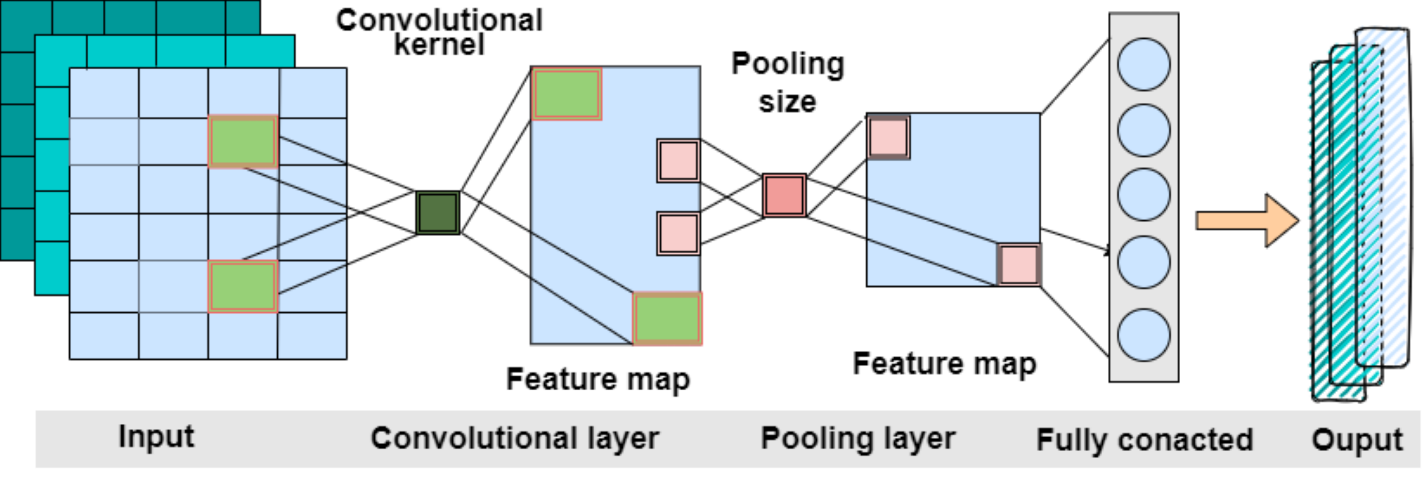


**Supplemental Figure 3:** CNN network structure

*
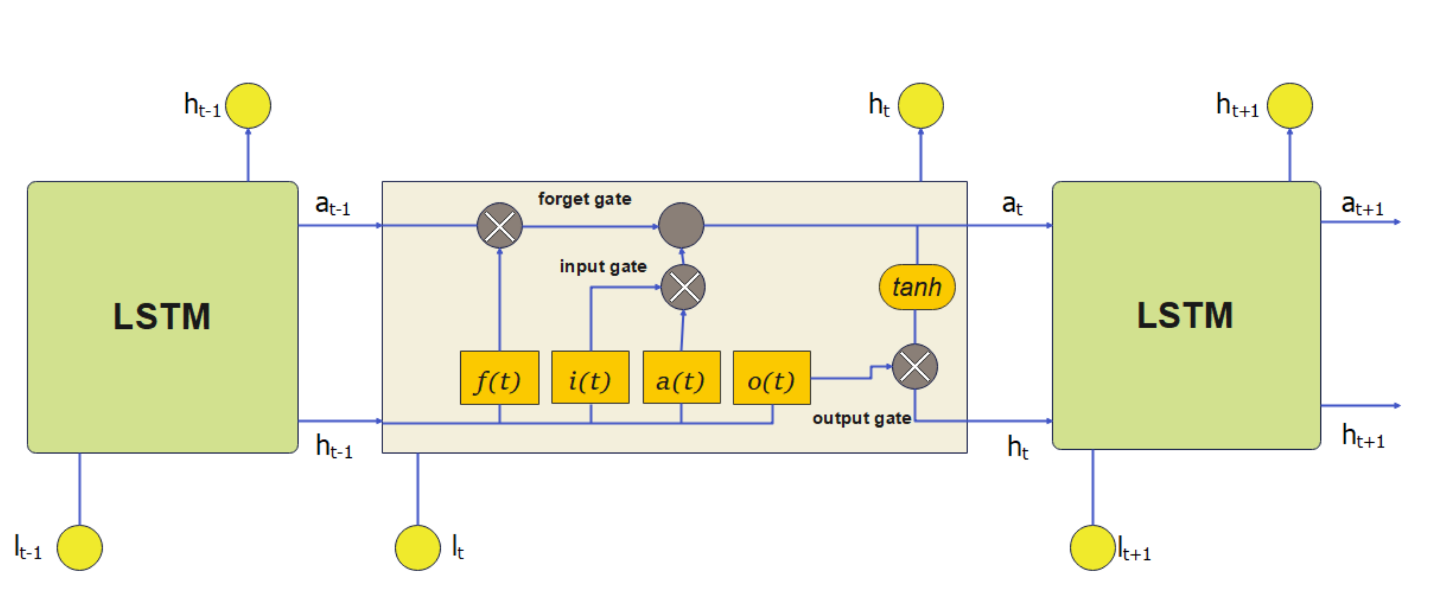
*

**Supplemental Figure 4:** LSTM network structure

| 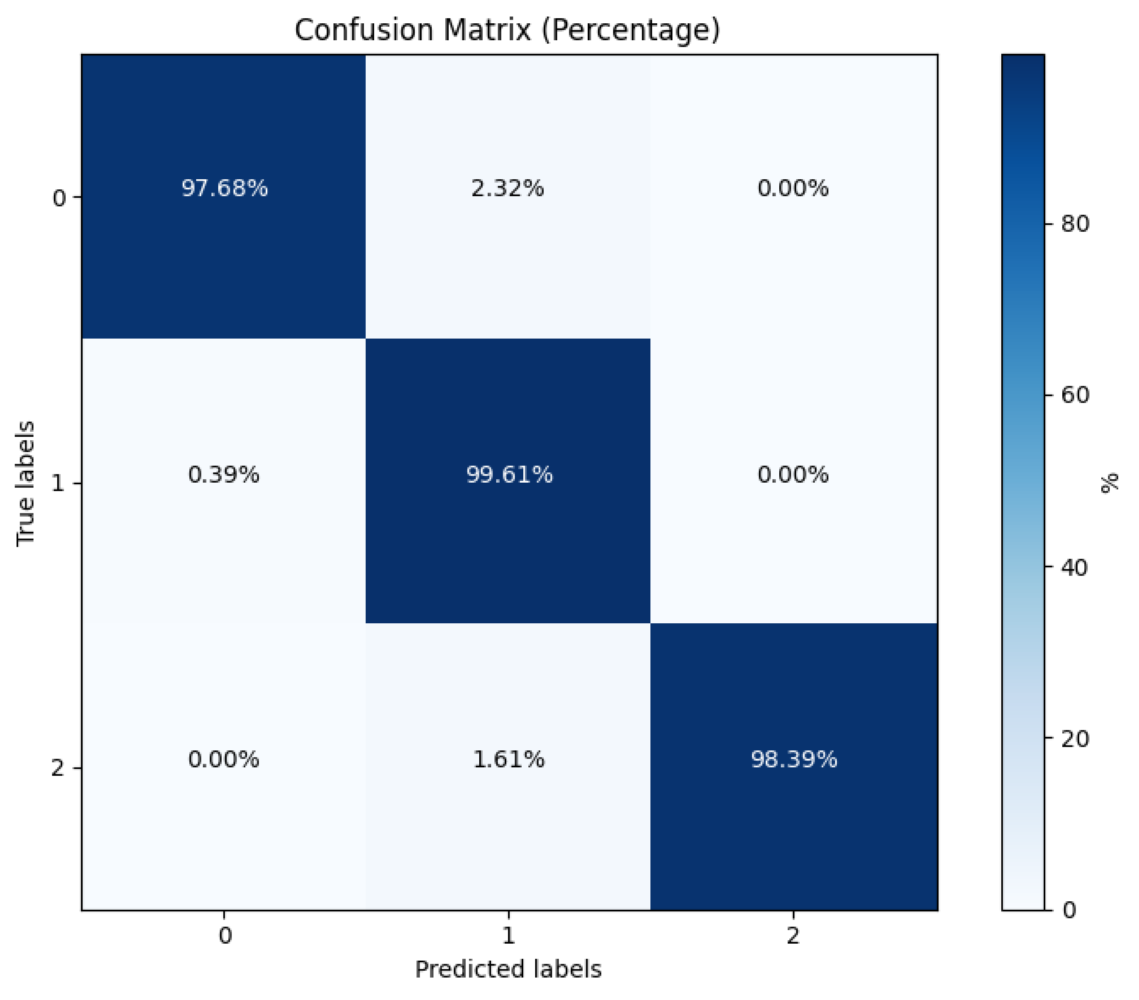 |
| --- |
| **Supplemental Figure 5:** Confusion matrix for the CNN-LSTM model on the dataset |

**
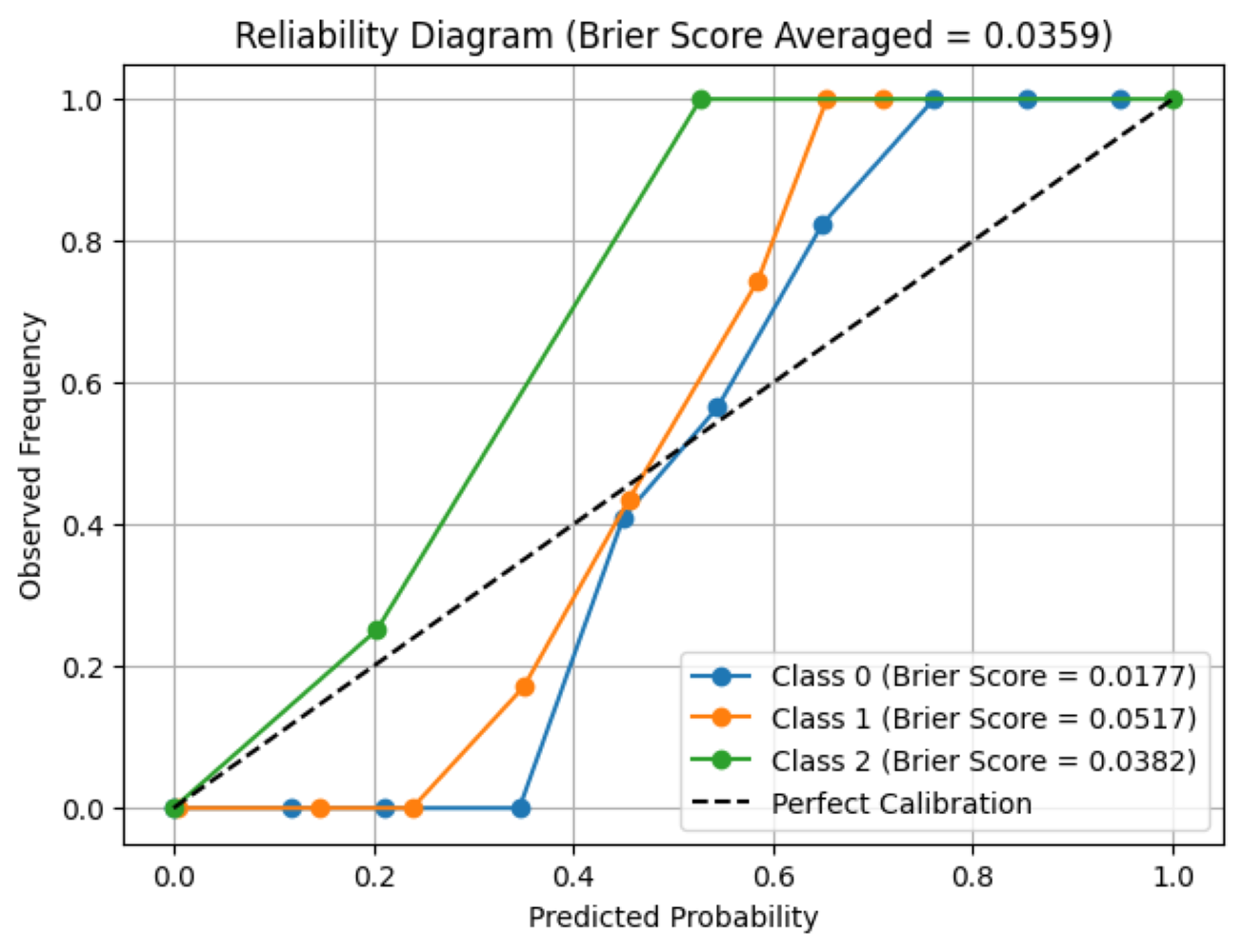
**

**Supplemental Figure 6:** Reliability diagram with Brier Scores

**
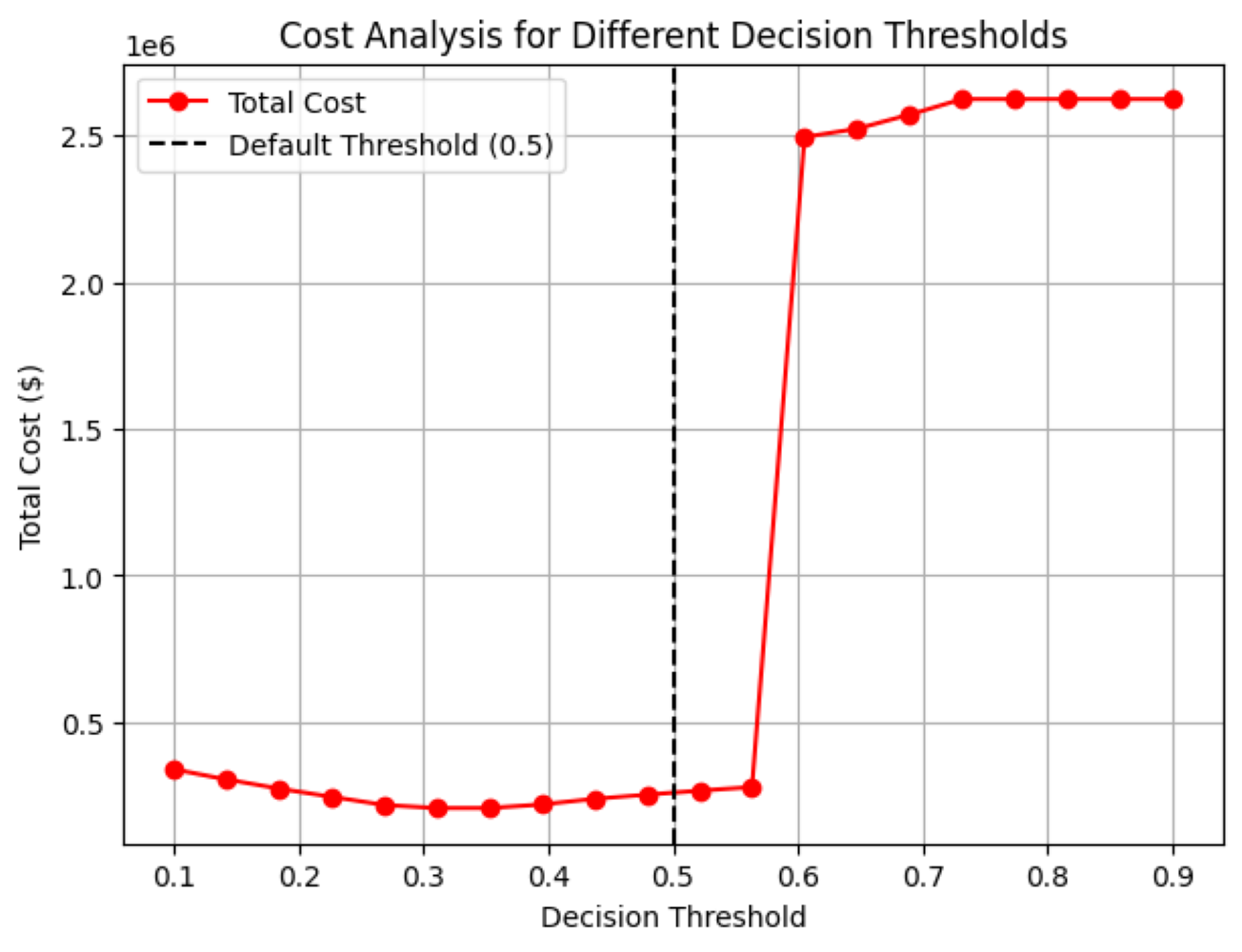
**

**Supplemental Figure 7:** Cost analysis for different decision thresholds


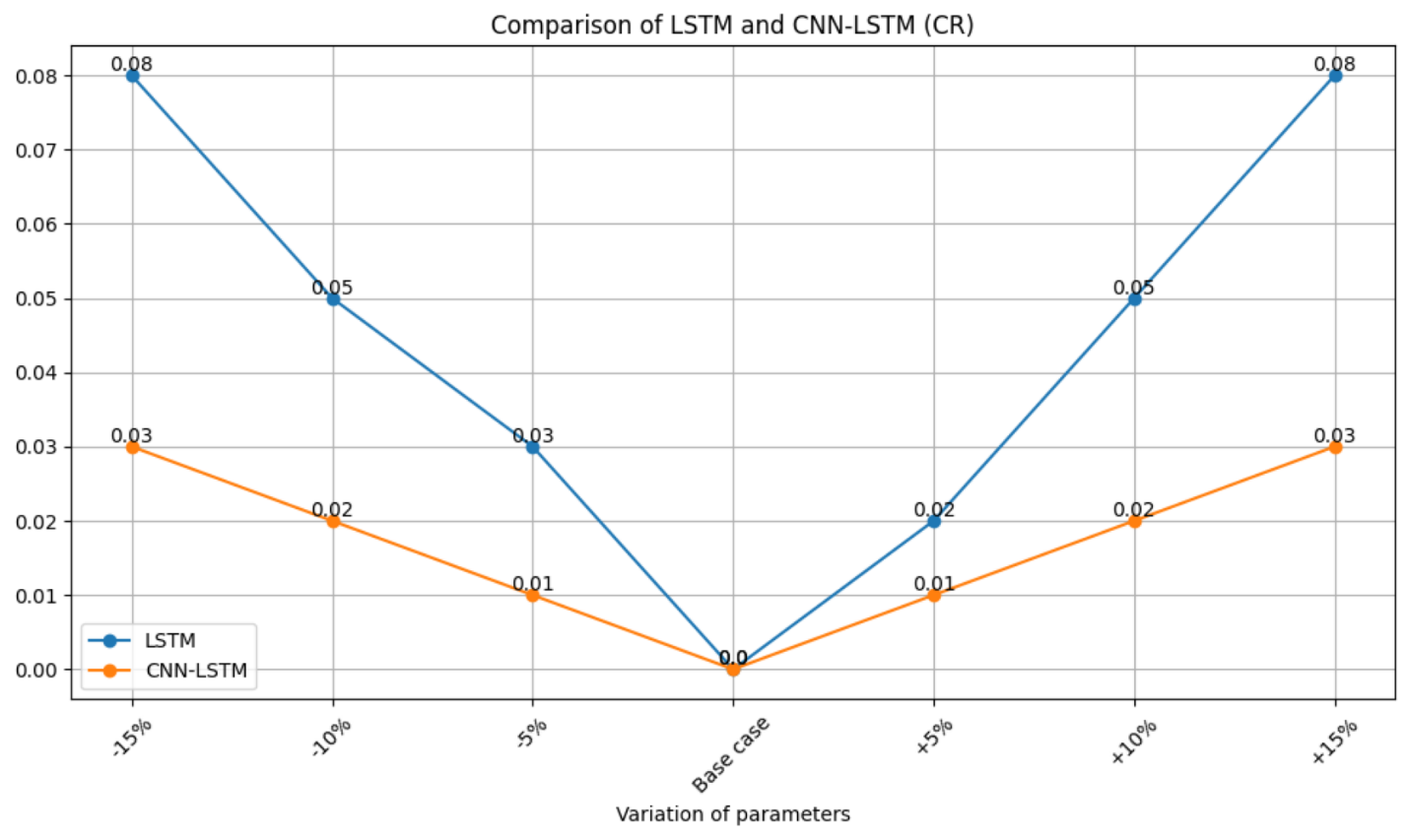


**Supplemental Figure 8:** Sensitivity analysis for CNN-LSTM and LSTM models, showcasing changes in CR


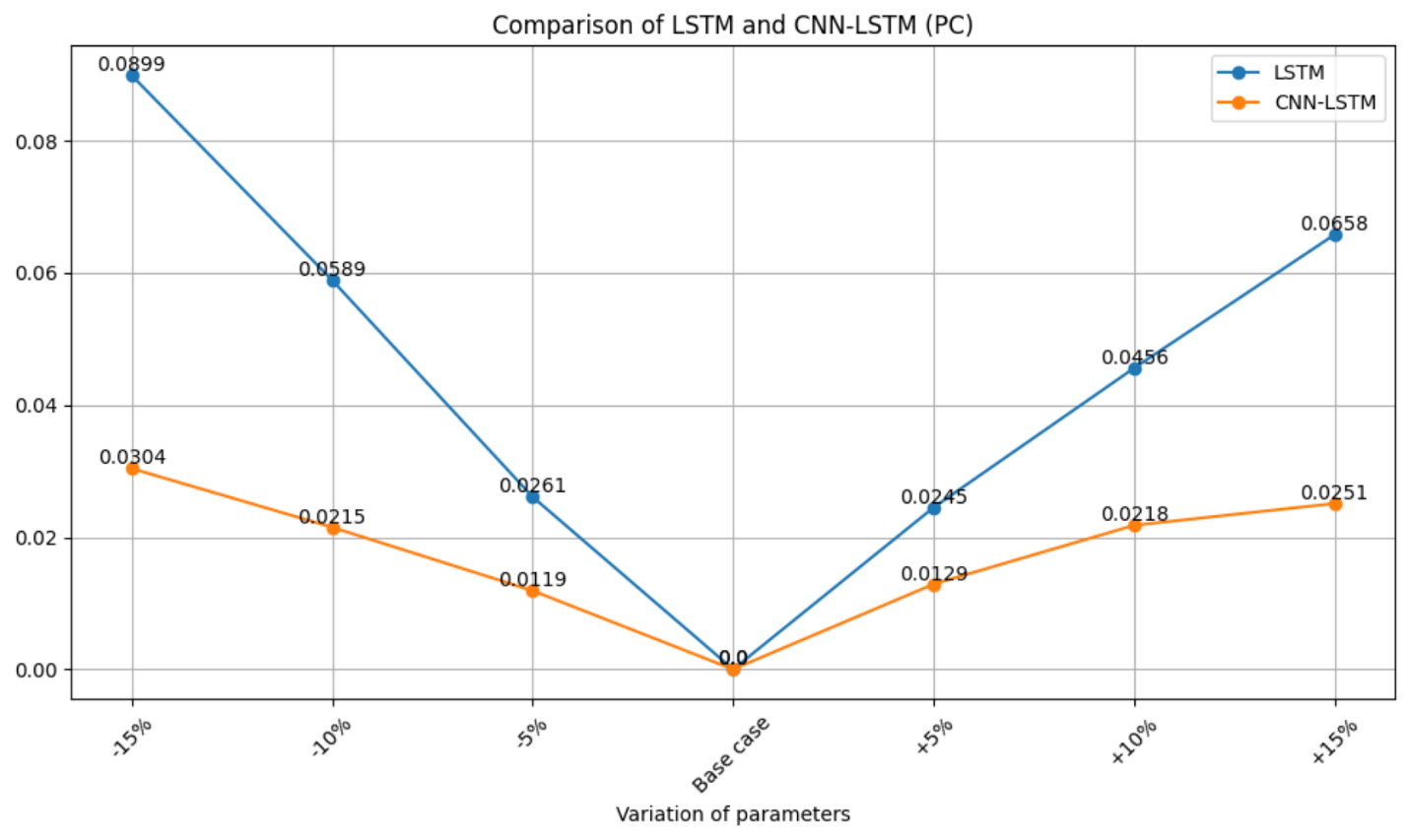


**Supplemental Figure 9:** Sensitivity analysis for CNN-LSTM and LSTM models, showcasing changes in PC

**Supplemental Table 1: Model hyperparameters**

| Hyperparameter | Value |
| --- | --- |
| Learning rate | 0.001 |
| Activation function | Relu |
| Loss function | Categorical cross-entropy |
| Dropout | 0.5 |
| Epoch | 100 |
| Batch size | 64 |
| Optimizer | Adam |

**Supplemental Table 2: Confusion matrix for classification**

|  | | Predicted claims | | |
| --- | --- | --- | --- | --- |
|  |  | Class 0 | Class 1 | Class 2 |
| Actual claims | Class 0 | True Positive (TP) | False Negative (FN) | False Negative (FN) |
|  | Class 1 | False Positive (FP) | True Negative (TN) | True Negative (TN) |
|  | Class 2 | False Positive (FP) | True Negative (TN) | True Negative (TN) |

**Supplemental Table 3: Comparison of claims prediction with other methods on Health Insurance Dataset India**

| DL methods | CNN-LSTM | LSTM | CNN | LogisticReg | GaussianNB | SVC | K-NN | CNN-GRU | CNN-BiLSTM |
| --- | --- | --- | --- | --- | --- | --- | --- | --- | --- |
| Accuracy | 97.2 | 90.5 | 93.7 | 94.2 | 79.5 | 84.2 | 62.3 | 91.1 | 92.5 |
| Precision | 97.5 | 90.7 | 94.1 | 94.5 | 81 | 84.3 | 63 | 92 | 92.8 |
| Recall | 97.2 | 89.9 | 93.4 | 94.2 | 79.5 | 84.2 | 62.3 | 91.1 | 92.5 |
| F1-Score | 97.2 | 90.5 | 93.7 | 94.4 | 78 | 84.1 | 62.7 | 91.5 | 92.6 |

**Supplemental Table 4: Sensitivity analysis results**

| Variation of parameters | CR | | PC | |  |
| --- | --- | --- | --- | --- | --- |
|  | LSTM | CNN-LSTM | LSTM | CNN-LSTM | |
| -15% | 0.0761 | 0.0298 | 0.0899 | 0.0304 | |
| -10 % | 0.0503 | 0.0225 | 0.0589 | 0.0215 | |
| -5 % | 0.0265 | 0.0129 | 0.0261 | 0.0119 | |
| Base case | 0 | 0 | 0 | 0 | |
| +5 % | 0.0241 | 0.0119 | 0.0245 | 0.0129 | |
| +10 % | 0.0516 | 0.0192 | 0.0456 | 0.0218 | |
| +15% | 0.0774 | 0.0251 | 0.0658 | 0.0251 | |
